# Investigation of the tissue equivalence of typical 3D-printing materials for application in internal dosimetry using monte carlo simulations

**DOI:** 10.1007/s13246-025-01532-2

**Published:** 2025-03-13

**Authors:** Ayse Karadeniz-Yildirim, Handan Tanyildizi-Kokkulunk

**Affiliations:** 1 Opticianry Program, Istanbul Aydın University, Florya, Bakirkoy, Istanbul, 34295 Turkey; 2 Radiotherapy Program, Altınbaş University, Kartaltepe Dist, Bakirkoy, Istanbul, 34147 Turkey

**Keywords:** PLA, ABS, Phantom, Internal dosimetry, Nuclear medicine

## Abstract

This study evaluates the dosimetric accuracy of PLA and ABS 3D-printed phantoms compared to real tissues using Monte Carlo simulations in radionuclide therapy. Materials and methods: A phantom representing average liver and lung volumes, with a 10 mm tumor mimic in the liver, was simulated for radioembolization using 1 mCi Tc-99 m and 1 mCi Y-90. The dose distribution (DD) was compared across PLA, ABS, and real organ densities. Results: For Tc-99 m, PLA showed a + 5.6% DD difference in the liver, and ABS showed − 35.3% and − 40.9% differences in the lungs. For Y-90, PLA had a + 1.7% DD difference in the liver, while ABS showed − 34.2% and − 34.9% differences in the lungs. Conclusion: In MC simulation, PLA is suitable for representing high-density tissues, while ABS is appropriate for simulating moderately low-density tissues.

## Introduction

In many radionuclide treatments applied in nuclear medicine clinics, patient-specific pre-dosimetry is performed by using only planar, SPECT/CT imaging or a combination of both [1]. Measurements on quasi-realistic anthropomorphic phantoms with a known activity concentration are a crucial requirement for accurate dosimetry [2, 3, 4]. Similarly, anthropomorphic phantoms are used in nuclear medicine imaging for differnet purposes, such as to determine the attenuation coefficient, to optimize the image quality for patients, suitable reconstruction parameters, evaluate scatter window width [5]. The goal of anthropomorphic phantoms, which are made of tissue-equivalent materials, is to create a realistic and accurate portrayal of the anatomy and the properties of tissues, organs, and the entire body for use in real and virtual investigations conducted in research and routine clinical duties [6]. Industrial production of these phantoms is costly and only financially viable when done in large quantities. Therefore, the validation of quantitative imaging and the associated absorbed dose estimation based on patient- or even just organ-specific geometries is hindered by the lack of commercially available phantoms, which typically consist of an arrangement of simple geometric objects like spheres and cylinders [7].

There are several technologies available to obtain the 3D printed product used in clinics for various investigations. A few of these methods are now commercially available thanks to 3D printing [7, 8], which makes it possible to produce phantoms on an individual basis with more unique geometries and presents a viable alternative to the manufacture of industrial phantoms. The three most popular varieties are stereolithography (SLA), fused filament fabrication (FFF), and fused deposition modeling (FDM), which are all types of jet technology. For the construction and testing of 3D printed phantoms and filaments, the bulk of studies published mention Polyjet and FFF/FDM as the preferred types [9]. Despite the fact that various materials have previously been produced that are appropriate for this activity and that Jet technologies are the most frequently employed in 3D printing phantom building as of 2018, FFF technology is the most widely used, accounting for 46% of all usage, with Jet technologies coming in third with 37% [10].

ABS (Acrylonitrile-butadiene-styrene) and PLA (Polylactic Acid) are the most commonly used 3D printing materials as a phantom in nuclear medicine, radiation oncology, and radiology clinics due to their versatility, ease of use, low cost, and similarity of densities to soft tissue [11, 12, 13]. The physical density values for PLA and ABS were reported to be 1.06 and 1.04 g/cm^3^, respectively [11]. Other materials including Photoluminescent PLA, PLA + Cu Woodfill, Bronzefill, Copperfill, PET, HIPS, TPU, TPE, PVA, Nylon, and other filaments were investigated less frequently [10].

In the field of radionuclide therapy, 3D printing technology is developing quickly. It is being used to produce implantable devices, medication delivery systems, and dosimetry phantoms tailored to individual patients. In this case, the main goals of using 3D printing are to reduce radiation exposure to healthy tissues, improve dose distribution, and improve treatment accuracy. Using patient imaging data, 3D-printed phantoms provide customized dosimetry in radionuclide therapy. Before therapy is administered, these phantoms allow for more precise dose calculations and distribution predictions due to their simulation of patient anatomy and tissue heterogeneity. Halloran et al. (2021) [14] has shown how useful 3D-printed phantoms are for accurately measuring radiation doses of therapeutic electron beams. Similarly, there are some studies reporting the use of polymer-based phantoms for dose verification [11, 12, 15].

Additionally, scaffolding for targeted radionuclide distribution and implantable radioactive seeds are made of 3D-printed materials. By controlling the discharge of radionuclides directly to the tumor site, these structures can be tailored to minimize radiation exposure from off-target sources. In their 2021 study, Mei et al. [16] investigated the application of radionuclide-loaded 3D-printed polymer-based scaffolds for localized therapy, observing notable enhancements in targeting accuracy.

3D-printed templates are used in brachytherapy to precisely position radioactive seeds inside malignancies. In 2022, Sohn et al. [17] looked at the use of 3D printing to customize brachytherapy templates. This allowed for more control over the distribution of doses, resulting in less radiation exposure to the surrounding tissues.

The development of biocompatible, 3D-printable materials that are safe for implantation and can contain radionuclides has also been the subject of recent study. The development of biodegradable polymers for implanted radioactive devices—which provide regulated radionuclide release and post-treatment bioresorption—was emphasized by Luca et al. in 2022 [18].

The goal of the study is to investigate virtually by Monte Carlo method to what extent the phantom made of PLA and ABS material reflects the dose distribution in real tissue in a radionuclide treatment.

## Materials and methods

In this study, radioembolization, which is one of the radionuclide treatments applied in nuclear medicine clinics, was preferred for internal dosimetry. In radioembolization treatment, it is known that the dose of tumor in the liver, liver parenchyma dose and lung dose are determined by dosimetry [19]. For this reason, the geometry of these three tissues is included in the study.

### Geometry

**Fig. 1:**
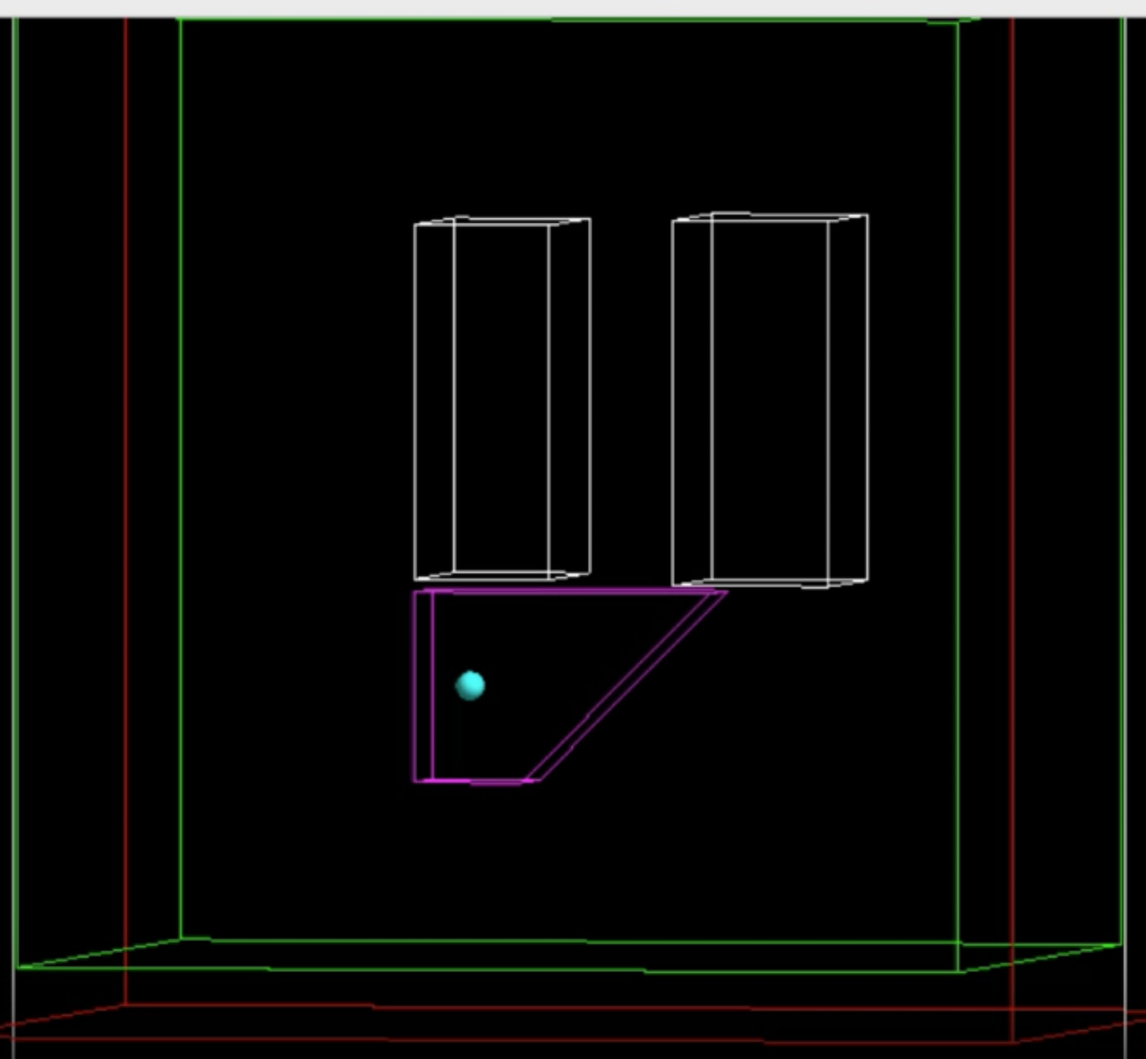
The visualization of virtual phantom geometry

All Monte Carlo simulations processes were executed on a computer with macOS Monterey Version 12.6.3, Core i5, 2.7 GHz, 8 GB, 1867 MHz, DDR3 memory and Intel Iris 6100 graphics processor. The preparation of simulation was started with creating the phantom geometry in GATE 8.1 version. The phantom geometry was created to include the average volume of liver and lung, and a spherical tumor imitation with a radius of 10 mm in the liver. In a water-filled 700 × 700 × 700 mm^3^ cube, all geometry was produced. The length (x), height (y), and depth (z), of the liver were specified in the wedge as being 220, 140, and 80 mm, respectively. Rectangles with dimensions of (100, 171, 261) mm and (116, 169, 269) mm, respectively, were used to establish the shape of the left and right lungs. The visual of the geometry used is given in Fig. 1.

**Fig. 2:**
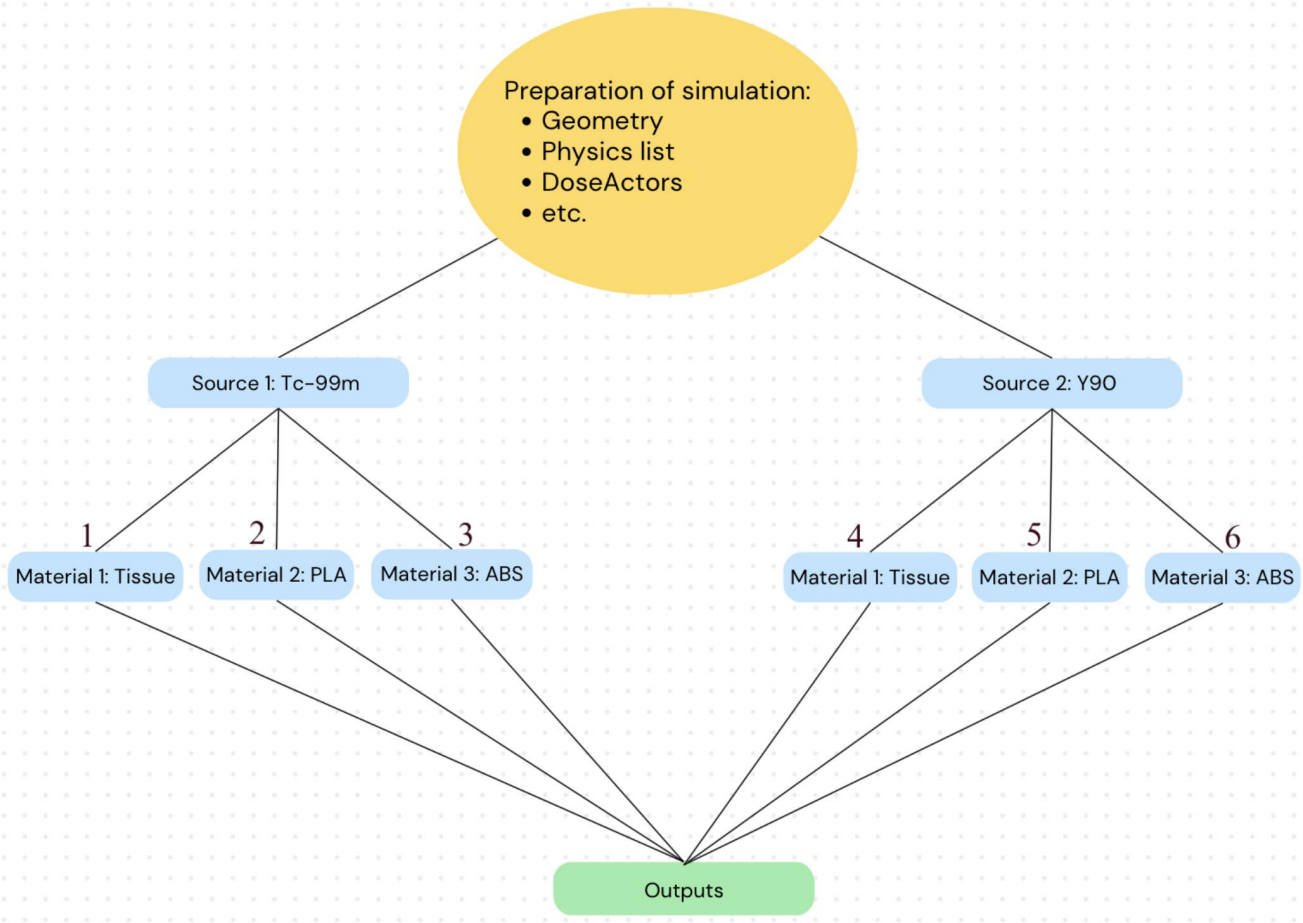
The simulation diagram according to radiation source and material variables

Since radionuclides that emit medium-energy gamma radiation are used in imaging and radionuclides that emit high-energy beta decay are used in treatment, two simulations were carried out by selecting a medium- and high-energy radionuclide. In the first simulation, Tc-99 m with 1 mCi activity was placed in the tumor mimic to determine imaging-based radiation doses (for medium energy) in the phantom, and in the second simulation, Y-90 with 1 mCi activity was placed in the tumor mimic to determine treatment-based radiation doses (for high energy) in the phantom. Before the simulation for both radionuclides, all tissue materials in the geometry were performed separately by defining PLA, ABS and true organ densities, respectively. Thus, the radiation doses of Tc-99 m and Y-90 radionuclides in real organs, PLA and ABS density phantoms were determined for comparison. The result was identified to be the generation of dose distribution graphs using ROOT and the acquisition of radiation doses for liver and lung mimics using C + + code. The steps describing the simulation are shown in Fig. 2 in detail.

### Define a new material

**Fig. 3:**
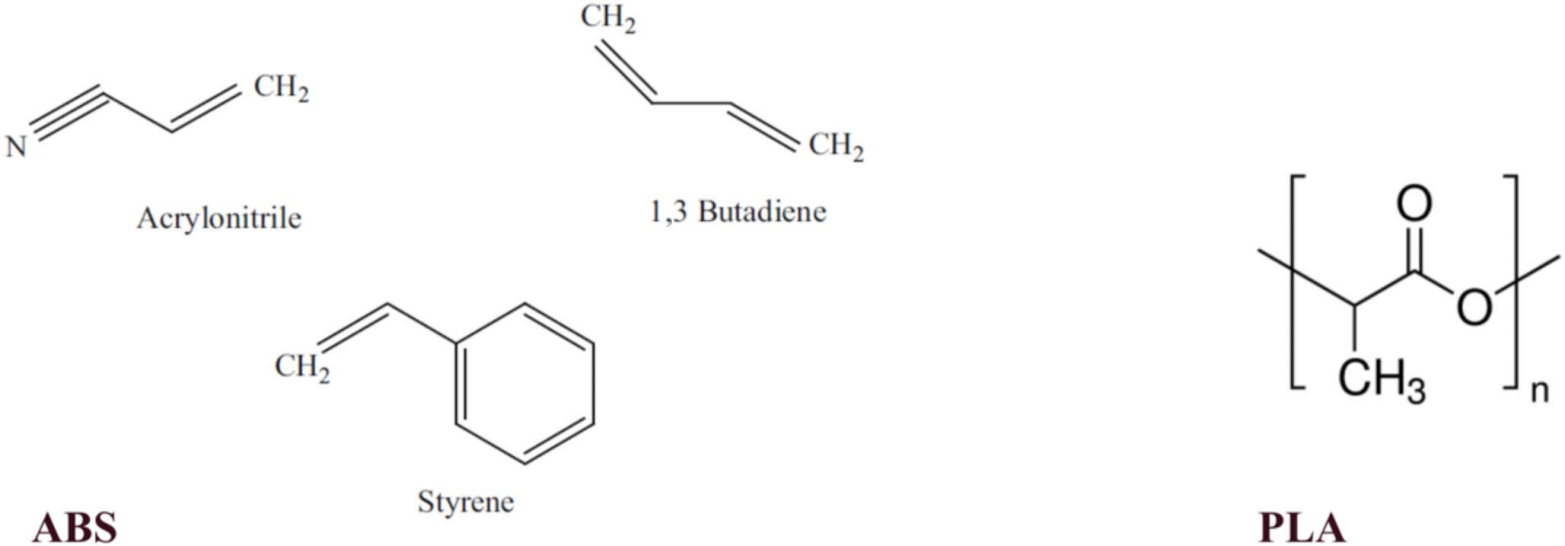
The diagram of the monomer units of ABS and molecular structures of PLA [21, 22]

Since the chemical and physical properties of PLA and ABS materials are not found in the material library named GateMaterials.db, it was added as a new material for this study. The material addition process was carried out for both materials by considering the density information that 1.04 g/cm^3^ for ABS and 1.24 g/cm^3^ for PLA [20], physical state of the material and the percentage of elemental compounds in the unit mass. The representation of the chemical structures of PLA and monomer units of ABS is given in Fig. 3 in detail.

### DoseActor

In the GATE simulation world, DoseActor acts as a sensitive detector capable of storing the energy stored in its added volume and interacting particle trace information. DoseActor divides a volume into 3D voxels (dosels) and records event information in those volumes. With the volume-weighted algorithm, DoseActor is calculated by dividing the total energy stored in the defined volume by the total volume and density of the volume material. DoseActor positions into right lung, left lung, and liver volumes were defined as (116, 1, 169), (100, 1, 171), and (220, 1, 80), respectively. The dose distributions of all volumes in 1 mm sections along the y-axis were calculated using the C + + analysis code. In addition to dose, the GATE doseactor command can also calculate dose squared and relative uncertainty in dose. The dose uncertainty of each voxel in this study was determined by multiplying the dose value accumulated in that voxel by the relative uncertainty (%) value that Monte Carlo computed for each voxel. The sum of all voxel values that were part of the volume and the average of all voxel uncertainties were used to determine dose values and uncertainties in total volumes.

### The physical processes and runtime in simulation

Finally, GATE8.1 is built on GEANT4 version 10.4.04. The physic-list builders have provide by the GEANT4 community and used “emstandard-opt3” for this simulation. Electromagnetic interactions (photoelectric event, compton scattering, anhillation etc.) and radioactive decay processes were chosen to create physical processes in simulation. Mersenne Twister algorithm was used as a random number generator. The simulation was run for approximately 30 h for Tc-99 m and approximately 17 h for Y-90 to simulate radioactive decay in 1 s. All macros were run consecutively for about 1 week to get all the data. The results were obtained as dose, uncertainty in dose, difference (percentage) in txt file.

## Results and discussion

Table 1 shows the absorbed doses per second and uncertainties in the virtual phantom. The absorbed doses and uncertainties are determined with the density of real tissue, PLA and ABS for Tc-99 m as a low energy radionuclide and Y-90 as a high energy radionuclide.

**Table 1:** Absorbed doses (Gy/s) and uncertainties obtained for critical organs by simulation using tissue, PLA and ABS materials for low energy (Tc-99 m) and high energy (Y-90)

	Tc-99 m					
	Liver		Right Lung		Left Lung	
	Absorbed doses (Gy/s)	Uncertainty	Absorbed doses (Gy/s)	Uncertainty	Absorbed doses (Gy/s)	Uncertainty
Tissue	7,16E-04	3,27E-06	4,60E-07	5,62E-09	9,65E-08	1,43E-08
PLA	7,56E-04	3,21E-06	2,91E-07	6,92E-09	5,31E-08	2,13E-09
ABS	7,58E-04	3,38E-06	2,98E-07	6,62E-09	5,71E-08	2,14E-09
	Y-90					
	Liver		Right Lung		Left Lung	
	Absorbed doses (Gy/s)	Uncertainty	Absorbed doses (Gy/s)	Uncertainty	Absorbed doses (Gy/s)	Uncertainty
Tissue	2,96E-03	2,02E-05	4,49E-07	2,02E-08	9,18E-08	7,93E-09
PLA	3,02E-03	2,02E-05	2,82E-07	1,16E-08	5,49E-08	3,81E-09
ABS	3,11E-03	2,24E-05	2,95E-07	1,22E-08	5,97E-08	4,21E-09

In the Tc-99 m simulation, the closest value to the tissue dose for the liver was calculated in PLA with 7.56E-04 ± 3.21E-06 Gy/s, and the closest values to the right and left lung dose were calculated in the ABS material phantom with 2.98E-07 ± 6.62E-09 and 5.71E-08 ± 2.14E-09 Gy/s, respectively. In the Y-90 simulation, the closest value to the tissue dose for the liver was calculated in PLA with 3.02E-03 ± 2.02E-05 Gy/s, and the closest values to the right and left lung dose were calculated in the ABS material phantom with 2.95E-07 ± 1.22E-08 and 5.97E-08 ± 4.21E-09 Gy/s, respectively. As a result of the general evaluation of the Tc-99 m and Y-90 simulations, it was found that the dose values of the ABS material phantom were closer to the dose values of the tissue material phantom, which is considered the reference.

**Fig. 4:**
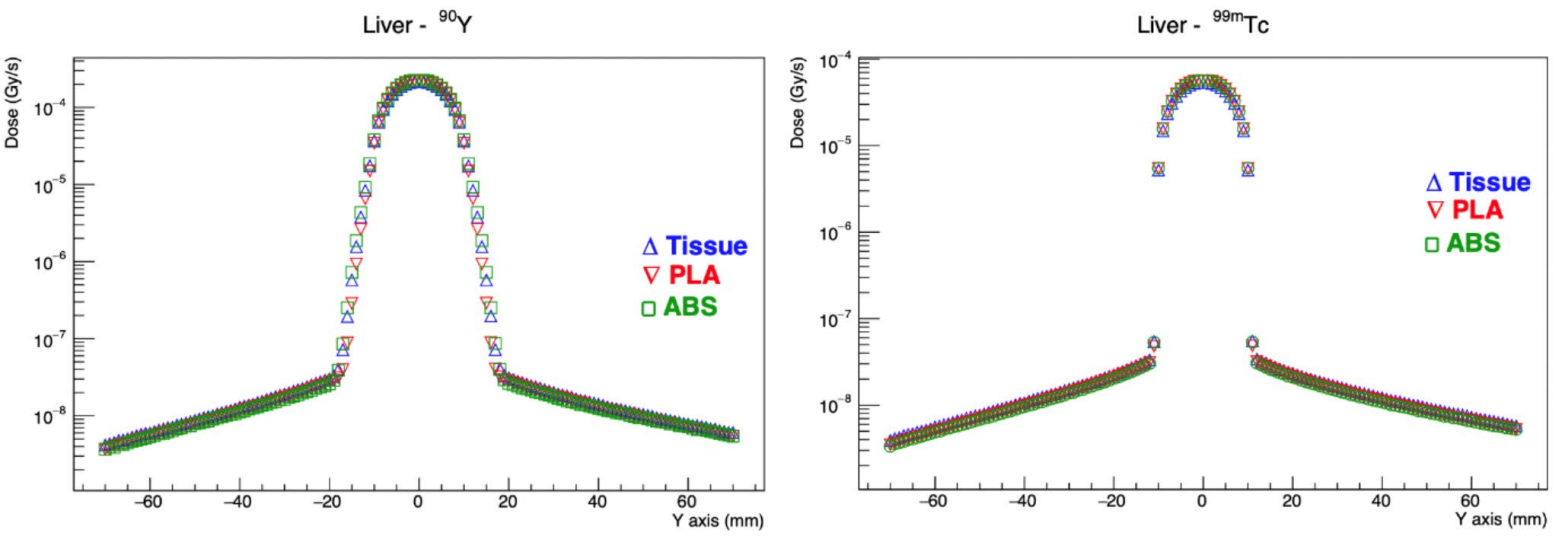
Dose distribution of Tc-99 m and Y-90 radionuclides in liver tissue depending on distance

**Fig. 5:**
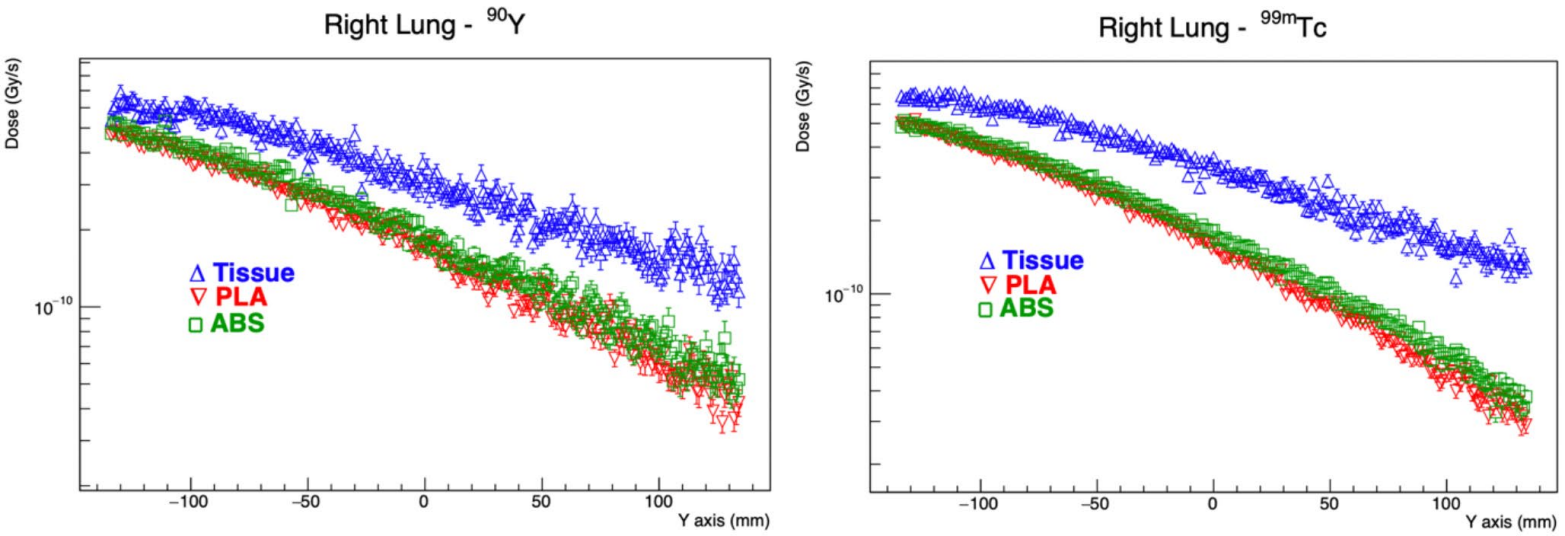
Dose distribution of Tc-99 m and Y-90 radionuclides in right lung depending on distance

**Fig. 6:**
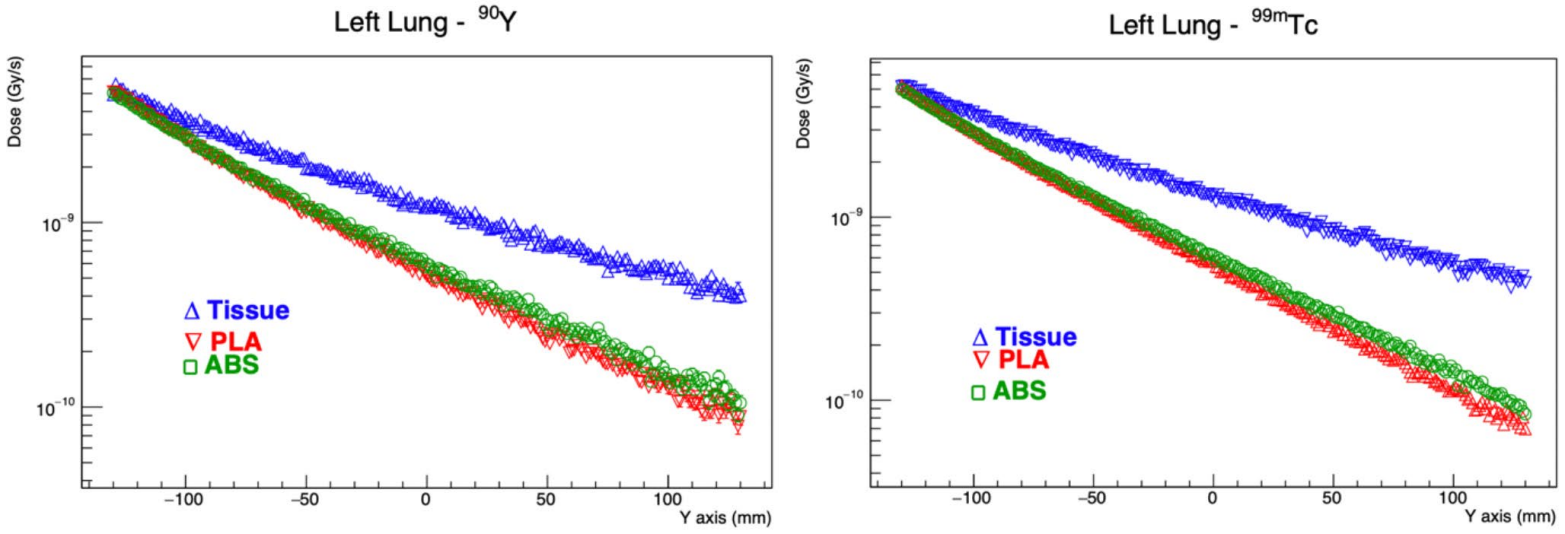
Dose distribution of Tc-99 m and Y-90 radionuclides in left lung depending on distance

Graphs showing dose distributions of Tc-99 m and Y-90 radionuclides in liver, right and left lung depending on tissue, PLA and ABS materials are shown in Figs. 4, 5 and 6.

Table 2 shows the absorbed dose differences (%) between PLA and ABS versus tissue.

**Table 2:** Absorbed dose differences (%) between PLA-Tissue and ABS-Tissue

	PLA-Tissue		ABS-Tissue	
	Difference		Difference	
	Tc-99 m	Y-90	Tc-99 m	Y-90
**Liver**	+ 5,6%	+ 1,7%	+ 5,9%	+ 4,7%
**Right Lung**	-36,8%	-37,8%	-35,3%	-34,2%
**Left Lung**	-45,04%	-40,2%	-40,9%	-34,9%

In the Tc-99 m and Y-90 simulation, it was shown that the dose values for the liver containing the tumor mimic rose by 5.6% and 1.7%, respectively, in comparison to normal tissue doses. Nevertheless, PLA was found to be the phantom substance that produced outcomes that were most accurate to reality. In the Tc-99 m simulation, the dose values for the right and left lung, which were situated away from the tumor, the radioactive source, were determined to be 35.3% and 40.9%, respectively, and 34.2% and 34.9% in the Y-90 simulation. When compared to tissue doses, ABS was found to be the phantom material that produced results that were most accurate when it came to distance from the source.

It is seen that the percentage of difference in doses increases considerably with distance from the source in the liver. Given that medical research often has a 95% level of confidence [23], the most ideal measurement system is validation based on the use of high-energy radionuclide, independent of the phantom material, and the comparison of doses close to the source. Studies in the literature that indicate dose distributions as a result of radiotherapy irradiation employing several ABS material phantoms support this conclusion [11, 12, 15].

Kumar et al. (2009) [11] used the IMRT technique to apply 6 MV X-ray radiation to an ABS-based phantom and then compared the dose distribution to that of normal tissue. They discovered that the dose distributions of the two phantoms varied by 2.63%. In a different investigation, Kairn et al. (2015) compared the inserts from the commercial tissue characterisation phantom with the cylindrical 3D printed phantoms’ film measures. They verified that for the eight clinical photon and electron radiation treatment beams employed in this investigation, the attenuation and scattering effects of the 3D printed samples were comparable. The difference between the measured and planned doses was determined to be less than 10% [12]. Similarly, in a study in which dose distributions were investigated by irradiating with ^137^Cs and performing OSL dosimetry, it was reported that the dose distribution difference was less than 0.5% for both ABS and PLA material phantoms [15].

Molecular radionuclide dosimetry verification using 3D phantoms has been the subject of multiple recent papers in the literature [24, 25, 26]. It was reported that the doses absorbed by the lesion and normal liver were compatible with the doses of the model patient in a dosimetry analysis using the AdboMan phantom composed of plexiglass (PMMA) material [24, 25]. According to a different study [26], the average dosages of a human 3D-printed phantom were between + 3 and − 53.1 in comparison to genuine tissue. The PLA-tissue or ABS-tissue dose changes in this investigation were determined to be within a more dependable range than those reported in the literature.

This study employed high-energy Y-90 as an example of radionuclide therapies and Tc-99 m as an example of medium-energy radionuclides used in nuclear medicine imaging. It was shown that PLA material produced more accurate findings when examining the dose distribution in the tissue/organ containing the radioactive source in the phantom, regardless of the radionuclide’s energy level (middle or high). It was discovered that using ABS material produced doses that were more in line with reality when examining the dose distribution in tissues and organs that were separated from the radioactive source.

The use of a high-energy radionuclide rather than a low-energy radionuclide like Tc-99 m will boost the accuracy of the results when validation is carried out with a phantom made of PLA or ABS material that is readily available in the clinic. Because a lesser dose difference was shown in the study to be generated by high-energy radionuclides. Additionally, since there will be less uncertainty in the doses, running the simulation with higher activity quantities will produce more accurate and trustworthy results. Due to the restricted computer power used for simulation, the sole limitation of this work is that Monte Carlo computations were performed with low activity.

Based on the absorbed dose differences presented in Table 2, PLA material exhibits dose values that are closer to those of liver tissue, as indicated by the smaller percentage differences in both Tc-99 m and Y-90 compared to the reference tissue. In contrast, ABS material demonstrates a closer resemblance to lung tissue, as seen in the more pronounced negative percentage differences, particularly for the left and right lungs. These findings suggest that PLA is more suitable for liver tissue modeling, whereas ABS is more representative of lung tissue in radiation dose simulations.

Furthermore, according to reports and articles based on internal dosimetry, including those from the ICRU and MIRD, dose uncertainties exhibit variation but are generally considered to fall within the range of 10–30% [27, 28, 29, 30]. This finding aligns with the uncertainty values presented in Table 1 of this study.

This study has several limitations, including computational power for simulation, low activity utilization, material accuracy, energy dependence and the use of specific radionuclides. The constrained computational power of this study resulted in low activity levels being used during the Monte Carlo simulations, which is one of its main shortcomings. This might have added further uncertainty to the dose estimates, especially for low-energy radionuclides like Tc-99 m. Second, the activity values used in the study’s dose estimates were comparatively low. Raising the activity could yield more accurate results, particularly for high-energy radionuclides such as Y-90, where measurements of the dose distribution could become more dependable and there would be less uncertainty. Thirdly, there are still limitations in the precision of the ABS and PLA materials employed in the study to replicate the precise radiological properties of human tissue [31], even if they demonstrated dose patterns that were similar to those of genuine tissue. There might be small differences between the real tissue and the phantom materials, which could affect the dose distributions, especially farther away from the source. Fourt, the study showed that the accuracy varied according to the energy of the radionuclide (high-energy Y-90 vs. medium-energy Tc-99 m). The inability of the phantom materials to reflect both kinds of energies at the same time may limit the results’ application to other energy ranges utilized in medical imaging and treatment. Finally, the study solely looked at Tc-99 m and Y-90 as radionuclides. The conclusions’ applicability for more comprehensive radionuclide dosimetry applications may be limited by the possibility that they will not hold true for additional radionuclides with distinct energy profiles or physical characteristics.

As known, only MC simulation results are presented in our study. No evaluation was made on physical, i.e. printed phantoms. In the literature, in printed phantoms, of course, density can be played with low, high, solid infill. For this reason, it should be known that phantoms printed from various materials can be used for different tissues/organ based on HU values [32].

Future research should focus on utilizing high-performance computing resources, such as GPU-based Monte Carlo simulations, to model higher activity levels with greater precision. This approach would improve the accuracy of dose calculations and reduce uncertainties. Additionally, expanding the study to include a wider range of radionuclides, such as Lu-177 and Ac-225, will provide deeper insights into the behavior of phantom materials across different therapeutic and diagnostic applications. To better mimic human tissue characteristics, future work should involve the development and characterization of novel 3D-printed materials with tunable properties, including density, attenuation coefficients, and scattering behavior. Investigating composite materials that incorporate radiopaque or radiolucent additives could further enhance tissue equivalence.

For validation, incorporating in vivo dosimetry techniques, such as thermoluminescent dosimeters (TLDs) or optically stimulated luminescent dosimeters (OSLDs), could provide a more direct comparison between simulated and actual clinical dose distributions. Additionally, benchmarking the simulation results against real patient treatment plans using commercial treatment planning systems would further validate the accuracy of the phantom-based dose calculations.

Given the growing role of theranostics in nuclear medicine, future studies should also explore the feasibility of using ABS and PLA phantoms in treatment planning and dose verification for theranostic agents. Assessing their performance in these contexts will help determine their suitability for emerging medical technologies, ultimately supporting advancements in both personalized therapy and imaging.

## Conclusion

By contrasting actual tissue measurements with computer simulations of dose distribution, this study offers a fresh strategy for enhancing radiation therapy. It optimizes treatment regimens by modeling dose absorption and interactions across tissues using techniques such as Monte Carlo simulations. More accurate and individualized treatment is made possible by the research’s quantitative relationship between expected doses and biological effects.

The study’s conclusions have important therapeutic ramifications for radiation therapy that is tailored to each patient. Clinicians can better tailor treatments to improve tumor control while reducing toxicity and side effects by validating simulations with actual tissue data. This precision boosts confidence in pre-treatment simulations, lowers the danger of radiation overdose and related cancers, and aids in the planning of next clinical trials.

This study showed the dose distributions of phantoms made of different materials, such as PLA and ABS, utilized in clinics for internal dosimetry applications, based on low and high energy radioactive sources. The findings demonstrated that the most effective measurement method uses PLA-based phantom material and high-energy radionuclides to compare doses near the source. Lastly, unless there is an extreme clinical necessity, ABS material phantoms should be used for internal dosimetry applications rather than PLA in the overall evaluation. It has been discovered that ABS tissue and material have more complimentary dose distributions. Future investigations involving nuclear medicine imaging or dosimetry can validate the results presented in this journal.

## Data Availability

If readers would like GATE codes, they can e-mail the authors. Readers will be given access to codes.

## References

[1] Ljungberg M, Sjögreen Gleisner K (2016) Personalized dosimetry for radionuclide therapy using molecular imaging tools. Biomedicines 4(4):25. 10.3390/biomedicines4040025 28536392 10.3390/biomedicines4040025 PMC5344265

[2] Ljungberg M, Celler A, Konijnenberg MW et al (2016) MIRD pamphlet 26: joint EANM/MIRD guidelines for quantitative 177Lu SPECT applied for dosimetry of radiopharmaceutical therapy. J Nucl Med 57(1):151–162. 10.2967/jnumed.115.159012 26471692 10.2967/jnumed.115.159012

[3] Dewaraja YK, Ljungberg M, Green AJ et al (2013) SNMMI MIRD Committee, MIRD pamphlet No. 24: Guidelines for quantitative 131I SPECT in dosimetry applications. J Nucl Med.;54(12):2182–8; 10.2967/jnumed.113.122390 10.2967/jnumed.113.122390 PMC3970715 24130233

[4] Dewaraja YK, Frey EC, Sgouros G et al (2012) MIRD pamphlet 23: quantitative SPECT for patient-specific 3-dimensional dosimetry in internal radionuclide therapy. J Nucl Med 53(8):1310–1325. 10.2967/jnumed.111.100123 22743252 10.2967/jnumed.111.100123 PMC3465844

[5] Kasraie N, Robinson A, Chan S (2018) Construction of an Anthropomorphic Phantom for Use in Evaluating Pediatric Airway Digital Tomosynthesis Protocols. Radiol Res Pract. Apr 18, 2018, 3835810; 10.1155/2018/3835810 10.1155/2018/3835810 PMC5932438 29850245

[6] Winslow JF, Hyer DE, Fisher RF et al (2009) Construction of anthropomorphic phantoms for use in dosimetry studies. J Appl Clin Med Phys 10(3):195–204. 10.1120/jacmp.v10i3.2986 19692982 10.1120/jacmp.v10i3.2986 PMC5720556

[7] Tran-Gia J, Schlögl S, Lassmann M (2016) Design and fabrication of kidney phantoms for internal radiation dosimetry using 3D printing technology. J Nucl Med 57(12):1998–2005. 10.2967/jnumed.116.178046 27445291 10.2967/jnumed.116.178046

[8] Durham M (2003) Rapid stereolithography, prototyping selective laser sintering, and polyjet. Adv Mater Processes 161:40–42

[9] Filippou V, Tsoumpas C (2018) Recent advances on the development of phantoms using 3D printing for imaging with CT, MRI, PET, SPECT, and ultrasound. Med Phys 45(9):e740–760. 10.1002/mp.13058 29933508 10.1002/mp.13058 PMC6849595

[10] Savi M, Andrade MAB, Potiens MPA (2020) Commercial filament testing for use in 3D printed phantoms. Radiat Phys Chem 174:108906. 10.1016/j.radphyschem.2020.108906

[11] Kumar R, Sharma D, Deshpande S et al (2009) Acrylonitrile butadiene styrene (ABS) plastic-based low cost tissue equivalent Phantom for verification dosimetry in IMRT. J Appl Clin Med Phys/Am Coll Med Phys 11:3030. 10.1120/jacmp.v11i1.3030 10.1120/jacmp.v11i1.3030 PMC5719786 20160681

[12] Kairn T, Crowe S, Markwell T (2015) Use of 3D printed materials as Tissue-Equivalent phantoms. Home World Congress Med Phys Biomedical Eng 51:728–731. 10.1007/978-3-319-19387-8_179

[13] Alssabbagh M, Tajuddin A, Abdul Manap M et al (2017) Evaluation of 3D printing materials for fabrication of a novel multi-functional 3D thyroid Phantom for medical dosimetry and image quality. Radiat Phys Chem 135. 10.1016/j.radphyschem.2017.02.009

[14] Halloran A, Newhauser W, Chu C, Donahue W (2021) Personalized 3D-printed anthropomorphic phantoms for dosimetry in charged particle fields. Phys Med Biol.;66(22). 10.1088/1361-6560/ac3047. PMID: 34654002 10.1088/1361-6560/ac3047 34654002

[15] Villani D Jr, Mascarenhas OR (2021) Study on electronic equilibrium of 137Cs gamma radiation for 3D printed phantoms using OSL dosimetry. J Phys: Conf Ser 1826(1):012057. 10.1088/1742-6596/1826/1/012057

[16] Mei Y, He C, Gao C, Zhu P, Lu G, Li H (2021) 3D-Printed degradable Anti-Tumor scaffolds for controllable drug delivery. Int J Bioprinting 7(4):418. 10.18063/ijb.v7i4.418 10.18063/ijb.v7i4.418 PMC8600306 34805597

[17] Sohn JJ, Polizzi M, Richeson D, Gholami S, Das IJ, Song WY (2022) A novel workflow with a customizable 3D printed vaginal template and a direction modulated brachytherapy (DMBT) tandem applicator for adaptive interstitial brachytherapy of the cervix. J Clin Med 11(23):6989. 10.3390/jcm11236989 36498563 10.3390/jcm11236989 PMC9738087

[18] Luca MD, Hoskins C, Corduas F, Onchuru R, Oluwasanmi A, Mariotti D, Conti B, Lamprou D (2022) 3D printed biodegradable multifunctional implants for effective breast cancer treatment. Int J Pharm 629:122363. 10.1016/j.ijpharm.2022.122363 36336202 10.1016/j.ijpharm.2022.122363

[19] Gulec SA, Mesoloras G, Dezarn WA et al (2007) Safety and efficacy of Y-90 microsphere treatment in patients with primary and metastatic liver cancer: the tumor selectivity of the treatment as a function of tumor to liver flow ratio. J Transl Med 5:15. 10.1186/1479-5876-5-15 17359531 10.1186/1479-5876-5-15 PMC1845138

[20] Bulavskaya A, Cherepennikov Y, Grigorieva A et al (2020) Theoretical study of the dose measurements reliability with longitudinally arranged dosimetry films in materials with different densities. J Inst 15(03):C03037. 10.1088/1748-0221/15/03/C03037

[21] Cuiffo MA, Snyder J, Elliott AM et al (2017) Impact of the fused deposition (FDM) printing process on polylactic acid (PLA) chemistry and structure. Appl Sci 7(6):579. 10.3390/app7060579

[22] Olivera S, Muralidhara HB, Venkatesh K et al (2016) Plating on acrylonitrile–butadiene–styrene (ABS) plastic: a review. J Mater Sci 51(8):3657–3674. 10.1007/s10853-015-9668-7

[23] Attia A, EVIDENCE-BASED MEDICINE CORNER (2005) Why should researchers report the confidence interval in modern research? Middle East Fertility Soc J 10:78

[24] Gear JI, Cummings C, Craig AJ et al (2016) Abdo-Man: a 3D-printed anthropomorphic Phantom for validating quantitative SIRT. EJNMMI Phys 3(1):17. 10.1186/s40658-016-0151-6 27495914 10.1186/s40658-016-0151-6 PMC4975728

[25] Ungania S, D’Arienzo M, Mezzenga E et al (2022) A workflow for dosimetry of 90Y radioembolization based on quantitative 99mTc-MAA SPECT/CT imaging and a 3D-Printed Phantom. Appl Sci 12(20):10541. 10.3390/app122010541

[26] Adam DP, Grudzinski JJ, Bormett I et al (2022) Validation of Monte Carlo 131I radiopharmaceutical dosimetry workflow using a 3D-printed anthropomorphic head and neck Phantom. Med Phys 49(8):5491–5503. 10.1002/mp.15699 35607296 10.1002/mp.15699 PMC9388595

[27] Stabin MG (2008) Uncertainties in internal dose calculations for radiopharmaceuticals. J Nuclear Med May 49(5):853–860. 10.2967/jnumed.107.048132 10.2967/jnumed.107.048132 18413398

[28] Stabin MG (2008) Radiopharmaceuticals for nuclear cardiology: radiation dosimetry, uncertainties, and risk. J Nucl Med 49:1555–1563 18765586 10.2967/jnumed.108.052241

[29] International Commission on Radiological Protection (ICRP) (1987) Radiation dose to patients from radiopharmaceuticals. Pergamon, Oxford, U.K. ICRP publication 53 3505163

[30] Loevinger R, Budinger T, Watson E (1991) MIRD primer for absorbed dose calculations. Rev. Society of Nuclear Medicine, ed. New York, NY

[31] Iftekar SF, Aabid A, Amir A et al (2023) Advancements and limitations in 3D printing materials and technologies: A critical review. Polymers 15(11):2519. 10.3390/polym15112519 37299318 10.3390/polym15112519 PMC10255598

[32] Tino R, Leary M, Yeo A et al (2019) Gyroid structures for 3D-printed heterogeneous radiotherapy phantoms. Phys Med Biol 64:21NT05. 10.1088/1361-6560/ab48ab 31561246 10.1088/1361-6560/ab48ab

